# Neuropsychological and neuroanatomical phenotype in 17 patients with cystinosis

**DOI:** 10.1186/s13023-019-1271-6

**Published:** 2020-02-26

**Authors:** Aurore Curie, Nathalie Touil, Ségolène Gaillard, Damien Galanaud, Nicolas Leboucq, Georges Deschênes, Denis Morin, Fanny Abad, Jacques Luauté, Eurielle Bodenan, Laurent Roche, Cécile Acquaviva, Christine Vianey-Saban, Pierre Cochat, François Cotton, Aurélia Bertholet-Thomas

**Affiliations:** 1 Service de neuropédiatrie Hôpital Femme Mère Enfant, Hospices Civils de Lyon, Institut des Sciences Cognitives Marc Jeannerod, CNRS UMR 5304, 67 boulevard Pinel, 69675 Bron, France; 2 Institut des Sciences Cognitives Marc Jeannerod, CNRS UMR 5304, L2C2, Bron, France; 3 Faculté de médecine Lyon Est, Université Claude-Bernard Lyon 1, Lyon, France; 4 EPICIME-CIC 1407/Inserm, UMR5558, Université de Lyon, Hospices Civils de Lyon, Bron, France; 5 Service de neuroradiologie, Hôpital Pitié-Salpêtrière, AP-HP, Paris, France; 6 Service de neuroradiologie, Centre Hospitalier Universitaire de Montpellier, Montpellier, France; 7 Service de néphropédiatrie, Hôpital Robert-Debré, AP-HP, Paris, France; 8 Service de néphrologie et diabétologie pédiatrique, Service de pédiatrie I, Centre Hospitalier Universitaire de Montpellier, Montpellier, France; 9 Service de rééducation fonctionnelle, Hôpital neurologique, Hospices Civils de Lyon, Bron, France; 10 Service de biostatistiques, Hospices Civils de Lyon, Bron, France; 11 Service maladies héréditaires du métabolisme et dépistage néonatal, Centre de Biologie et Pathologie Est, Groupement Hospitalier Est (GHE), Hospices Civils de Lyon, Bron, France; 12 Centre de référence des maladies rénales rares – Néphrogones – Filière ORKiD, Bron, France; 13 Service de radiologie, Centre Hospitalier Lyon-Sud, Hospices Civils de Lyon, Pierre Bénite, France; 14 CREATIS, CNRS UMR5220, INSERM U1044, Université Lyon 1, INSA Lyon, Villeurbanne, France

**Keywords:** Cystinosis, Neuroimaging, Neuropsychological profile

## Abstract

**Background:**

Cystinosis is a rare autosomal recessive disorder caused by intracellular cystine accumulation. Proximal tubulopathy (Fanconi syndrome) is one of the first signs, leading to end-stage renal disease between the age of 12 and 16. Other symptoms occur later and encompass endocrinopathies, distal myopathy and deterioration of the central nervous system. Treatment with cysteamine if started early can delay the progression of the disease. Little is known about the neurological impairment which occurs later. The goal of the present study was to find a possible neuroanatomical dysmorphic pattern that could help to explain the cognitive profile of cystinosis patients. We also performed a detailed review of the literature on neurocognitive complications associated with cystinosis.

**Methods:**

17 patients (mean age = 17.6 years, [5.4–33.3]) with cystinosis were included in the study. Neuropsychological assessment was performed including intelligence (Intelligence Quotient (IQ) with Wechsler’s scale), memory (Children Memory Scale and Wechsler Memory Scale), visuo-spatial (Rey’s figure test) and visuo-perceptual skills assessments. Structural brain MRI (3 T) was also performed in 16 out of 17 patients, with high resolution 3D T1-weighted, 3D FLAIR and spectroscopy sequences.

**Results:**

Intellectual efficiency was normal in patients with cystinosis (mean Total IQ = 93). However the Perceptual Reasoning Index (mean = 87, [63–109]) was significantly lower than the Verbal Comprehension Index (mean = 100, [59–138], *p* = 0.003). Memory assessment showed no difference between visual and verbal memory. But the working memory was significantly impaired in comparison with the general memory skills (*p* = 0.003). Visuospatial skills assessment revealed copy and reproduction scores below the 50th percentile rank in more than 70% of the patients. Brain MRI showed cortical and sub-cortical cerebral atrophy, especially in the parieto-occipital region and FLAIR hypersignals in parietal, occipital and brain stem/cerebellum. Patients with atrophic brain had lower Total IQ scores compared to non-atrophic cystinosis patients.

**Conclusions:**

Patients with cystinosis have a specific neuropsychological and neuroanatomical profile. We suggest performing a systematic neuropsychological assessment in such children aiming at considering adequate management.

## Introduction

Cystinosis is a generalized lysosomal storage disease caused by intralysosomal cystine accumulation, leading to cellular dysfunction of many organs. It is a rare autosomal recessive disorder related to mutations in the *CNTS* gene located on 17p13 and encoding for a protein (cystinosin), which is a transport carrier for cystine across the lysosomal membrane. Once in the cytoplasm, cystine (the disulfide amino acid cysteine) is reduced to cysteine. In cystinosis, this transport out of lysosomes is defective and leads to intralysosomal cystine accumulation and progressive tissue damage [1]. It is interesting to note that cystine accumulation may begin very early, likely during fetal life [2].

The first symptoms result of a severe proximal tubulopathy (renal Fanconi syndrome) and begin between 6 months and 1 year including anorexia, vomiting, polyuria and failure to thrive [2, 3]. Renal injury leads to end-stage renal disease (ESRD) between the age of 12 and 16 [4]. Initiating cysteamine therapy before 5 years of age was shown to decrease the incidence and delay the onset of ESRD [4]. Corneal cystine crystals are usually visible (using slit lamp examination) after the first year of life, and photophobia appears around 2 years of age. Other symptoms occur later and encompass endocrinopathies (diabetes and hypothyroidism), distal myopathy and encephalopathy.

Thanks to renal transplantation and cysteamine therapy, cystinosis patients now live well into adult life. It is thus very important to better understand long-term complications such as neurocognitive ones, which impact their quality of life. Several neurological complications may occur in NC patients including: (i) *distal progressive myopathy* (25 to 50% in adult large series [4, 5]), (ii) *swallowing dysfunction* in more than half of the adult patients (with an abnormal oral, pharyngeal and esophageal phases of swallowing in 24, 51 and 73% of patients respectively [6]), (iii) *cerebral atrophy* [7–9], (iv) *cystinosis encephalopathy* with mental deterioration, cerebellar and pyramidal signs [7], (v) *seizures* [8], (vi) *stroke* [7, 10, 11], (vii) *idiopathic intracranial hypertension* [12–14] and (viii) *Chiari malformation* [15]. Furthermore, cystinosis patients have an overall intelligence within the normal range, but impairments in visual processing, visual memory and visual motor coordination, poor executive functions and arithmetic skills [16–21].

A few neuropathological descriptions have been performed in cystinosis patients [11, 22–24]. Cerebral atrophy, small cerebellum with decreased cerebellar cellularity, multifocal cystic necrosis, focal dystrophic calcification, multifocal patchy demyelination of the white matter, spongiform change and vacuolization of both the cerebral cortex and white matter [22–24]. The analysis of cystine content per tissue revealed high cystine levels in the basal ganglia, medulla, pons, dura and choroid plexus [22, 24]. Cystine cristals were observed within the cytoplasm of pericytes and parenchymal brain cells (probably oligodendrocytes) [24]. Neutel et al. reported a patient with recurrent ischemic strokes caused by intracranial stenosis [11]. Interestingly, Berger et al. reported a cystinosis patient with a cervical myelopathy. A stereotactic biopsy revealed cystine crystal deposition and an intense vasculopathy affecting small and medium sized blood vessels [25]. Moreover, a *Ctns*^−/−^ mice model of cystinosis study suggested that cystinosis-associated central nervous system complications are likely due to progressive cystine accumulation [26].

The present study is part of a longitudinal French study entitled « A cohort of patients with cystinosis: compliance to cysteamine and neurological complications » (Hospital Clinical Research Program *CrYSTobs*). We present here the developmental trajectory, neuropsychological and neuroanatomical phenotype of 17 cystinosis French patients, using for the first time a 3 T MRI scanner (which increases the signal/noise ratio), as well as their renal status. The goal of the present study was to find a possible neuroanatomical dysmorphic pattern that could help to explain the cognitive profile of cystinosis patients. We also performed for the first time a detailed and exhaustive review of all the studies describing cognitive profile or brain MRI in cystinosis patients.

## Patients and methods

### Participants

#### Recruitment procedures

Recruitment was accomplished through the French Network of pediatric nephrologists (*Société de Néphrologie Pédiatrique*) and within the 3 reference centres for rare renal diseases that participated in the study (Paris, Montpellier and Lyon). This study was approved by the Ethical Committee of our institution (Comité de Protection des Personnes Lyon-Sud Est II, 2010–030-2, 09/08/2010). After being informed about the aims of the study, all patients and their parents gave written informed consent before the study procedure started.

Age-matched child and adult healthy controls were recruited through local advertisements. Adult healthy control participants and the parents of each child included in the study signed an informed consent before the study procedure started.

#### Patients with nephropathic cystinosis

Seventeen patients with confirmed diagnosis of cystinosis (defined by clinical signs and leukocyte cystine level or genetic mutation (*n* = 15)) were included in the study (10 females and 7 males). 53% of the patients with an identified genotype were homozygous for the 57 kb *CTNS* deletion (8/15). Two patients were from consanguineous families. The patients mean age was 17.6 years (age range: 5.4 to 33.3 years). Seven were adults. All of them performed the neuropsychological assessment. Sixteen of them also performed the MRI assessment (mean age: 18.3 years, [7–33.6]).

#### Age-matched healthy controls

Sixteen age-and sex-matched healthy controls were included in the brain MRI study. Their mean age was 18.4 years (age range: 7.3 to 33.7 years). None of them met exclusion criteria: history of neurological or psychiatric disorder, repetition of a grade, learning disability requiring rehabilitation (speech therapy, psychomotor or oculomotor therapy). The exclusion criteria were chosen to be sure to include only typically developing children and none with a neurodevelopmental disorder. Healthy controls were recruited by posted flyers at the hospital sites and electronic postings (emails …) to subjects who participated to previous research studies as healthy controls.

### Clinical data

Clinical data were collected from the patients, their parents, as well as from the patient’s medical records, including: birth parameters, early development, language, school curriculum, age at diagnosis, age at start of cysteamine treatment, performance of a neuropsychological assessment prior to the inclusion in the present study, renal events (kidney transplantation, dialysis) and extra-renal complications. The following parameters were also recorded: body weight, height, head circumference, systolic and diastolic blood pressure, maximum walking distance, maximum walking time, and maximum number of floors they could climb. In addition to clinical data from the pediatric nephrology database, a detailed neurological assessment was performed in eight cystinosis patients, including handgrip strength assessment using an hydraulic hand dynamometer (JAMA).

### Neuropsychological assessment

***Intellectual functioning assessment*** was performed using age-appropriate Wechsler scales: WPPSI-III (Wechsler Preschool and Primary Scale of Intelligence) for children aged 2 years and 6 months to 6 years, WISC-IV (Wechsler Intelligence Scale for Children) for children aged from 6 to 16 years, and WAIS-IV (Wechsler Adult Intelligence Scale) for children above 16 years and adults. These scales are a standardized method to test the Intelligence Quotient (IQ) in both children and adults. In addition to the Total IQ, the four main indices were also analysed: Verbal Comprehension Index (a measure of verbal concept formation), Perceptual Reasoning Index (a measure of non-verbal and fluid reasoning), Working Memory Index, and Processing Speed Index. A WPPSI-III scale was used in only one child. As the WPPSI-III scale provides a Verbal and Performance Intelligence Quotient score, these two scores were substituted for Verbal Comprehension Index and Perceptual Reasoning Index respectively [21].

***Memory assessment*** was performed using the Children Memory Scale (CMS) for children aged from 5 to 16 years, and using the Wechsler Memory Scale (WMS-III) for children above 16 years and adults. This scale assesses both visual and verbal memory, immediate and delayed, and gives a score for general memory and a score for working memory.

***Visuo-spatial assessment*** was performed using the Rey–Osterrieth complex figure test (copy and reproduction from memory). Both scores (copy and reproduction from memory) were analyzed. The strategy used to perform the task was also assessed.

***Visuo-perceptual assessment*** was performed using the NEPSY scale for children aged between 3 and 12 years old.

### Brain MRI image acquisition

All structural brain MRI acquisitions were performed on 3 Tesla scanners. Three different scanners were used for the patients: a Philips scanner (3 T Achieva MR System, Philips Medical Systems, Best, Netherlands) in Lyon (*n* = 9), a General Electrics (GE Healthcare, Milwaukee, Wisconsin, USA) in Paris (*n* = 6) and a Siemens scanner (Siemens Medical Solutions, Erlangen, Germany) in Montpellier (*n* = 1). All the MRI images in aged-matched healthy controls were acquired in Lyon on two different scanners: a 3 T MR Philips scanner (3 T Achieva MR System, Philips Medical Systems, Best, Netherlands) for healthy controls who were age-matched to the patients included in Lyon; and a 3 T MR Siemens scanner for the other healthy controls. High resolution (0.9*0.9*0.9 mm) structural imaging with a 3D T1-weighted TFE (Turbo Field Echo) sequence (TR 6600, TE 2.9 ms, FOV 240*240) was obtained for each patient and sex and age-matched healthy control. In addition, a 3D FLAIR sequence was performed (FOV 250*250*180, voxel size: 1.1*1.1*0.6, TR 8000, TE 362, TI 2400).

### Brain MRI image analysis

MRI images were clinically reviewed by a neuroradiologist with 20 years of experience (FC) and a pediatric neurologist (AC). The following items were scored for each of the brain MRI for both patients and age- and sex-matched healthy controls: Evans’ Index (ratio of maximum width of the frontal horns of the lateral ventricles and maximal internal diameter of skull at the same level on axial MRI slice), brain atrophy (frontal, parietal, temporal, occipital, corpus callosum, cerebellum), FLAIR hypersignal (frontal, parietal, temporal, occipital, brain stem). Brain atrophy and FLAIR hypersignals for each brain region were rated as normal, or showing mild, moderate or severe abnormalities.

### Measurement of leukocyte cystine level

The leucocyte cystine level was determined for all the patients around the date of the MRI. White blood cells (WBC) were isolated from whole blood collected into a citric acid-citrate-dextrose (Bawden et al.) tube [27]. After lysis and deproteinization, cystine was measured using liquid chromatography-tandem mass spectrometry (LC-MS/MS, Api3200 – Applied Biosystems, Concord, Canada) [28]. Protein were measured using BiCinchoninic acid Assay (BCA) (commercial kit BC Assay Protein Quantitation kit Interchim, Montluçon, France) on an ABX Pentra 400 (HORIBA, Kyoto, Japan) analyser. Results are expressed in nmol hemicystine/mg of protein. Furthermore, as a single leucocyte cystine level does not provide enough information relative to average control of cystinosis, we also computed the mean of leucocyte cysteine level performed every 3 months during the year when the MRI and neuropsychological assessment were performed.

### Assessment of renal function

Glomerular filtration rate (GFR) was estimated using the Schwartz equation [29] for all the patients around the date of the MRI and/or neuropsychological assessment [30]. showed that the Schwartz equation was more reliable than the CKD-EPI equation for estimating GFR in children and adolescents and in adults with mild to moderate kidney impairment up to the age of 40 [30]. KDIGO stage was determined for each cystinosis patient according to the Clinical Practice Guideline for the Evaluation and Management of Chronic Kidney Disease (2012).

### Statistical analysis

Statistical analysis was performed using R software (http://www.r-project.org).

#### Natural history, developmental trajectory, and biological data

For continuous variables, results were expressed as mean within the cystinosis patient group and the range for this variable was given into brackets. Discrete variables were expressed as numbers of cases and percentages. Correlation between age at diagnosis and year of birth was analyzed using the Pearson test. Significance was considered at *p* < 0.05.

#### Neuropsychological data

Regarding IQ data, the normality of the data distribution was first checked using the Shapiro and Wilk normality test. The mean and standard deviation (SD) of the Total IQ were computed. As intellectual deficiency is considered for an IQ below 70, we also computed the number of patients having an IQ below 70. To further characterize the IQ distribution in cystinosis patients, we also reported the number of patients with low average IQ (70 < IQ < 90). To test for the effect of age at treatment on TIQ, we performed a linear regression analysis on TIQ scores with age at start of cysteamine. Significance was considered at *p* < 0.05. In an exploratory analysis, we analysed the IQ profile using paired t-tests Bonferroni corrected for the number of variables analysed. Significance was considered at *p* < 0.05.

Regarding the memory assessment, the normality of the data distribution was first checked using the Shapiro and Wilk normality test. The mean and standard deviation (SD) of the general memory score were computed. In an exploratory analysis, we analysed the memory profile using paired t-tests Bonferroni corrected for the number of variables analysed. Significance was considered at *p* < 0.05.

Regarding the visuo-spatial skills assessment, we computed the percentage of patients scoring below the 50th percentile in the copy and in the recall conditions. We also computed the number of patients having a pathological visuo-motor precision index.

Furthermore, as another exploratory analysis, we compared the IQ scores and the general and working memory between the adult group (defined as older than 14 years) and the children group (supplementary data).

#### Brain MRI study

Regarding the Evans’ index, the normality of the data distribution was first checked using the Shapiro and Wilk normality test. A between-group analysis was then performed between cystinosis and age-matched healthy control group using an ANOVA. A significance level of *p* < 0.05 was chosen.

The number of patients atrophic and with FLAIR hypersignals was computed in each group (cystinosis patients and age-matched healthy controls). A between group analysis was performed using a Fisher’s exact test.

Moreover, based on neuroimaging data, we defined two groups of cystinosis patients: an atrophic group (when the severity of the brain abnormalities were rated as moderate or severe) and a non-atrophic group (when the severity of the brain abnormalities were rated as normal or mild). An ANOVA was applied in the cystinosis group with one within group factors (atrophic / non atrophic) on the TIQ score. Significance was considered at *p* < 0.05.

### Review of the literature

We performed a systematic review of the literature, searching Medline/PubMed. We used the following search terms ‘nephropathic cystinosis’ AND ‘MRI’, ‘nephropathic cystinosis’ AND ‘neuroimaging’, ‘nephropathic cystinosis’ AND ‘cognition’. All studies published before November 2016 were considered for inclusion.

## Results

### Natural history of cystinosis patients

Pregnancy was uneventful for all patients. All patients but three were born full term; two of the three were born at 36 weeks of gestation and one at 33. Birth weight, height and head circumference were within the normal range. Neonatal history was normal for all patients.

The mean age at diagnosis was 2.0 years [0.3–6.5]. The mean age at start of cysteamine treatment was 2.9 years [1.3–9.7]. Three patients had reported extended periods without cysteamine therapy. The mean leukocyte cystine level was 1.05 nmol hemicystine/mg of protein [0.1–3.3] around the date of the MRI. The mean leukocyte cystine level during the year when the MRI was performed, was 1.1 nmol hemicystine/mg of protein [0.3–2.7]. All patients received cysteamine therapy, either immediate (*n* = 2) or delayed (*n* = 15) release form, with a mean dose of 1194 mg per day [550–1950], i.e. 31.2 mg/kg/day [15.1–58] or 948.5 mg/m^2^/day [522.4–1470.9]. The mean measure of the auto-assessment of observance over the last 3 months rated by cystinosis patients was 9.7/10 [8–10].

Cystinosis patient mean height was − 1.5 SD for their age [from − 3.8 to + 0.3]. Five out of 17 (29%) cystinosis patients had growth retardation and received growth hormone therapy. Their Body Mass Index (BMI) and their Head Circumference (HC) were within the normal range (mean BMI: − 0.2 SD [from − 1.5 to + 1.7]; mean HC: − 0.2 SD [from − 2.5 SD to + 2.2SD]). Out of the 17 patients, 4 (24%) had hypothyroidism, 3 (18%) had a gastrostomy,3 (18%) had diabetes mellitus, 4 (24%) had headaches, one (6%) had seizures, and 3 (18%) had depression. All patients were photophobic and one of them had corneal graft. All patients had ophtalmologic assessment, either before the neuropsychological assessment (*n* = 16) or a few months later (n = 1). Slit lamp examination revealed corneal cystin crystals only in 6.7% of the patients. No papillory oedema was noted in cystinosis patients. Visual acuity was performed in all cystinosis patients but two, either before the neuropsychological assessment or in the following months. Most patients had normal values (10/10th visual acuity in 13 patients, 9/10th in one). Two were visually impaired leading to the unability to perform the visuospatial tests (one had 5/10th visual acuity, the other one could not perform the assessment. She had marked photophobia too).

### Renal status

Renal transplantation had been performed in 65% of the patients, with the first kidney transplant done at a mean age of 13.5 years [8.4–18]. At inclusion, the mean graft survival was 10.6 years [0.3–28.8]. Eleven patients had one renal transplantation, two received two, and one patient three. 41% of the cystinosis patients received steroids when brain MRI was performed.

Their mean systolic and diastolic blood pressure was + 0.5 SD [from − 1.4 to + 3 SD, with only one patient above + 2 SD] and + 0.3 SD [from − 1.9 to + 2.9 SD, with 3 patients above + 2 SD], respectively. The mean estimated GFR in cystinosis patients was: 48.7 mL/min per 1.73 m^2^ [6.7–86.1]. The repartition of the estimated GFR regarding KDIGO staging was as follows: stage 1: 0%; stage 2: 35%; stage 3: 41%; stage 4: 12%; stage 5: 12%. More precisely, the mean creatinine clearance rate amongst native kidney cystinosis patient was 52.4 mL/min per 1.73m^2^ [30.5–75.2], whereas it was 59.3 mL/min per 1.73m^2^ [19.9–86.1] in transplanted kidney cystinosis patients and 13 mL/min per 1.73m^2^ [6.7–21.1] in hemodialysis cystinosis patients.

### Developmental trajectory of nephropathic cystinosis patients

Cystinosis patients hold their head at a mean age of 3.8 months [1.1–5.7], and sat alone without support at 7.3 months [5.5–9.5]. They walked alone at 17.8 months [12.4–22.4]. Six of them walked after 18 months (motor delay). The mean age at which utterance of the first words occurred was 13.9 months [8.4–22.4]. First words utterance was delayed in two patients (occurring respectively at 21.6 and 22.4 months). The mean age at producing sentences was 23.1 months [17.4–40.5].

Regarding school curriculum, they entered kindergarten at 3.1 years [3–4], elementary school at 6.1 years [6–7], and secondary school at 11.1 years [10–12]. 27% of the patients had to repeat grades (between once and four times). Only one patient out of 17 had to follow specialized education. Amongst the adult patients, 57% went to college.

Only 24% of the patients had benefitted from a neuropsychological assessment prior to the study. Each patient had an average of 1.9 siblings [0–4].

### Neuromuscular assessment

Six out of 17 cystinosis patients (35%) had myopathy with distal amyotrophy (hands amyotrophy). This might be underestimated as only half of the cystinosis patients were clinically assessed by a neurologist. Interestingly, we performed an objective muscular handgrip strength assessment using an hydraulic hand dynamometer (JAMA) on a subgroup of cystinosis patients. Dominant hand grip strength was 16.01 kg [4.7–29.7] in cystinosis patients as compared to 40.75 in controls [33.2–55.25]. Even two patients who were evaluated clinically as having normal muscular strength had decreased hand grip strength as measured with JAMA. 4/17 patients had weak voice with nasal intonation. 2/17 (11.8%) had swallowing impairment.

Half of cystinosis patients had no limitation regarding the maximum distance they could walk. The mean maximum distance the rest of them could walk was 3400 m [2000–4000]. Similarly, 50% of the patients had no limitation regarding the maximum time they could walk, whereas for the others the maximum walking time was 1.4 h [1–2]. The maximum number of floors they could climb was without limit for 50% of the patients, and a mean of 3 for the others [2–4].

### Neuropsychological assessment

Fifteen out of 17 cystinosis patients (88%) were right-handed.

#### Intelligence assessment

**Fig. 1:**
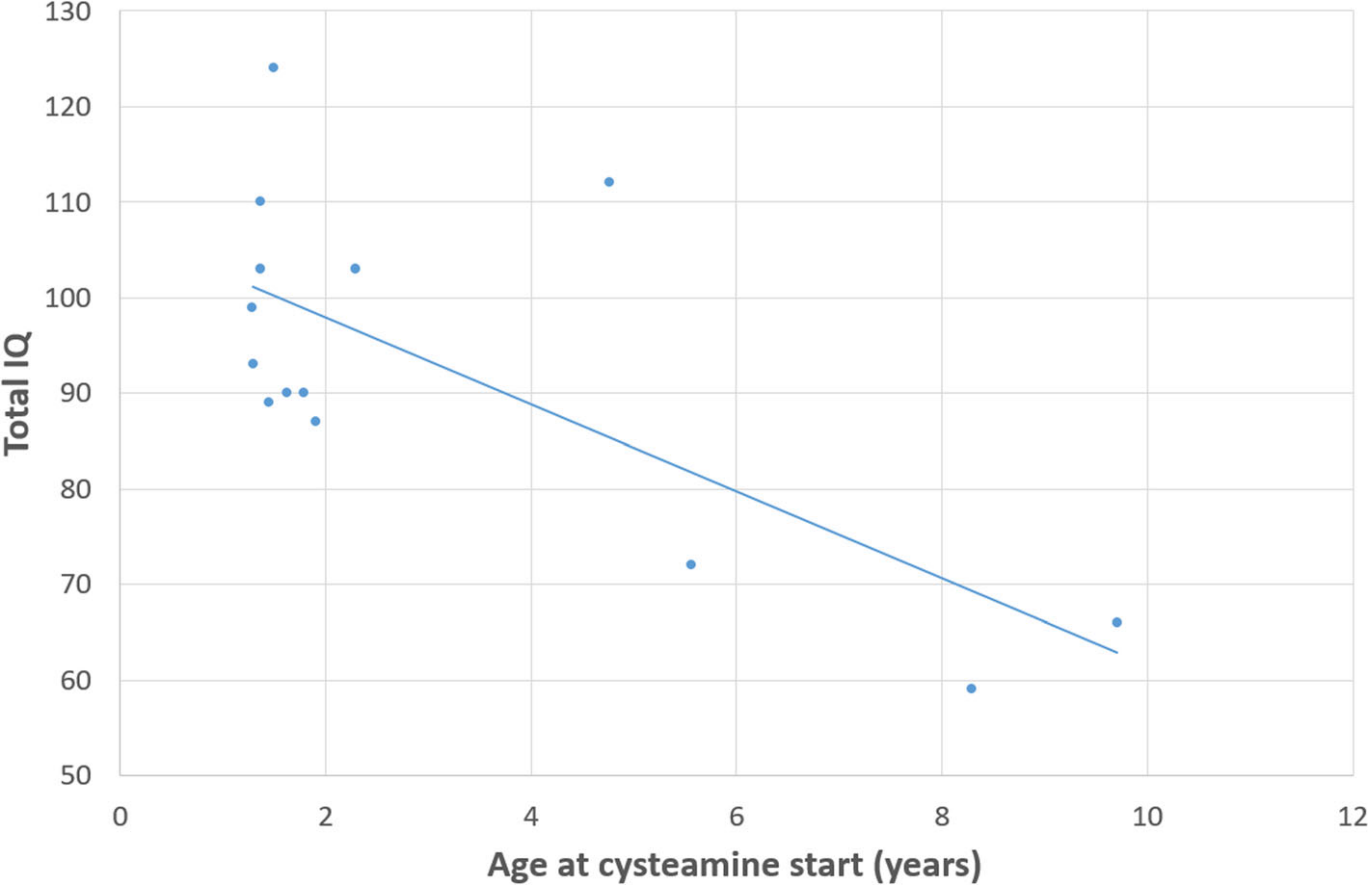
Correlation between Total IQ and age at cysteamine start (r^2^ = 0.5, *p* < 0.005)

Age-appropriate Wechsler scale was performed in all patients. However two patients could not perform the visual sub-test given their visual impairment. The mean total Intelligence Quotient (IQ) was 93 [59–124], SD = 18.1. Total IQ was not computed in the two patients who were visually impaired as they were unable to complete all the visual sub-tests. One of them had high average Verbal Comprehension Index (VCI), whereas the other one had pre-dementia. Two other patients scored below 70. Nevertheless, none of them was diagnosed as having ID, but with multiple specific learning disorder syndrome associated with lower socioeconomic status. According to DSM V criteria, a strong argument against ID was their good adaptive skills in both cases, including daily-life autonomy. Three other patients had a total IQ within the low average range (between 70 and 89). A significant correlation was found between Total IQ and the age at start of cysteamine (r^2^ = 0.5, *p* < 0.005, Fig. 1). More precisely, the sooner cysteamine was started, the higher the IQ was. It is interesting to note that all cystinosis patients who started cysteamine before 2 years of age had an IQ within the normal range (*n* = 11). There was no correlation between TIQ and patient age (r^2^ = 0.09, *p* = 0.75).

**Fig. 2:**
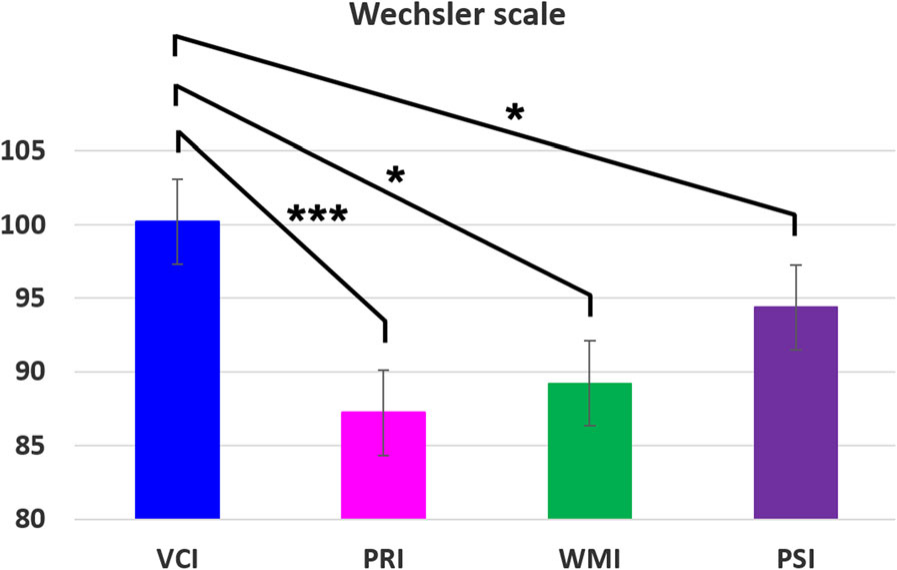
Wechsler scale in cystinosis patients (VCI: Verbal Comprehension Index; PRI: Perceptual Reasoning Index; WMI: Working Memory Index; PSI: Processing Speed Index). *: < 0.05; *** < 0.005

Figure 2 shows the results for each of the four indices. The Perceptual Reasoning Index (PRI) was significantly more impaired than the Verbal Comprehension Index (VCI) and the Processing Speed Index (PSI) (*p* = 0.003 and *p* = 0.03 respectively). The Working Memory Index (WMI) was significantly more impaired than the VCI (*p* = 0.04). None of the patients had a significantly higher PRI compared to VCI.

#### Memory assessment

The general memory score was within the normal range (mean = 102.2, SD = 16.9). There was no dissociation between visual and verbal memory. Working memory was significantly more impaired than the general memory (*p* = 0.003, Additional file 1: Figure S1). The immediate memory scored within the normal range.

#### Visuo-spatial skills assessment

Using the ReyOsterrieth complex figure test, we showed that 67% of cystinosis patients scored below the 50th percentile in the copy condition, and 80% in the recall condition. It is interesting to note that 50% of the patients used a parcellar strategy to perform the task: more precisely they used a juxtaposition of specific design elements rather than the overall figure.

Furthermore, sensory-motor skills assessment (from the NEPSY scale) was performed in children. 100% of patients had a pathological visuo-motor precision index.

### Brain MRI study

**Fig. 3:**
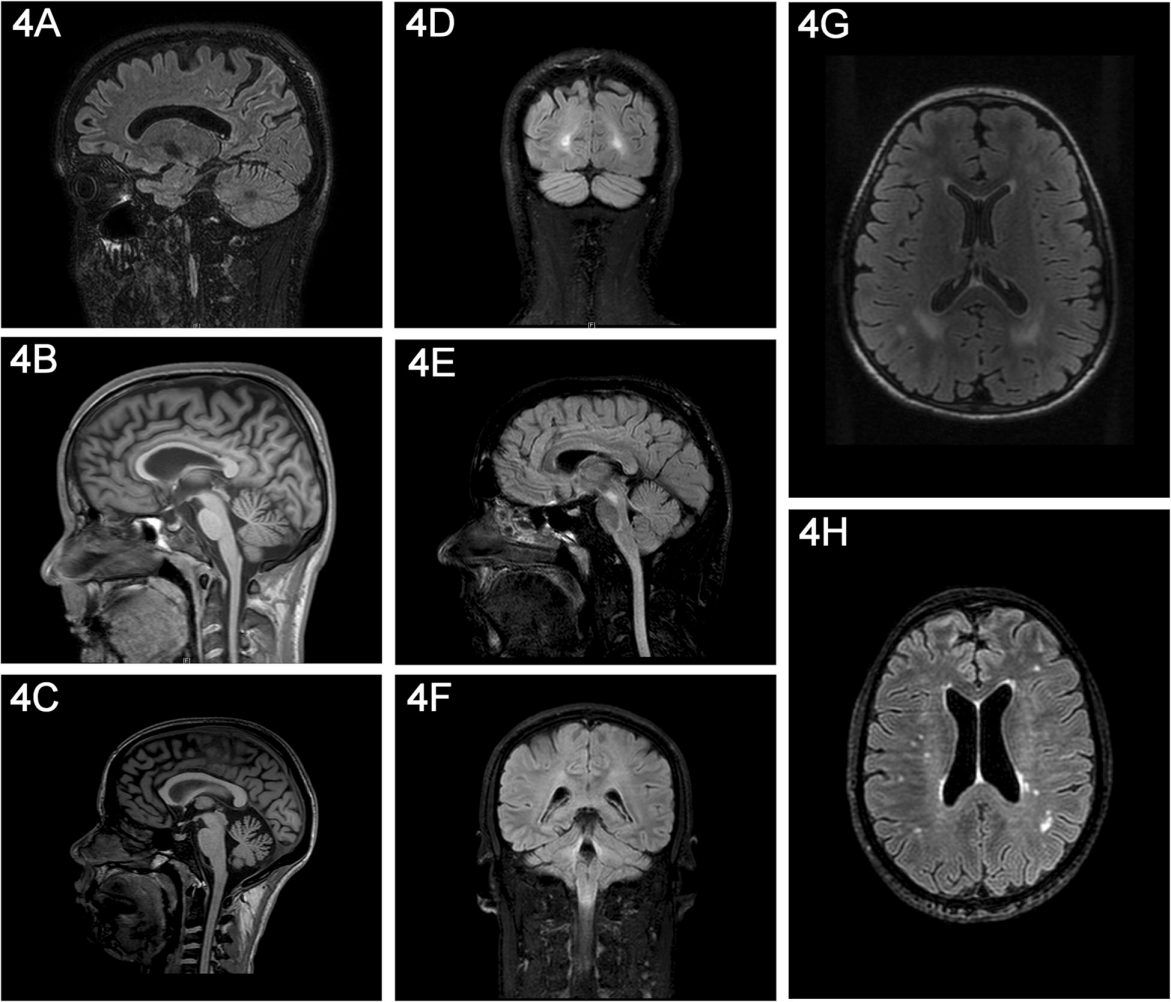
Brain MRI in cystinosis patients (**a**: parietal atrophy with meningeal hypersignal surrounding the precuneus; **b**: corpus callosum atrophy; **c**: vermis atrophy; **d**: occipital FLAIR hypersignals; **e**: ponto-mesencephalic FLAIR hypersignals; **f**: FLAIR hypersignals located around the 4th ventricle including cerebellar peduncles; **g-h**: more diffuse FLAIR hypersignals)

Table 1 and Fig. 3 show the results in both cystinosis patients and age- and sex-matched healthy controls. No healthy control and two cystinosis patients had a developmental venous anomaly (one in the frontal, the other one in the cerebellar region respectively). None of the cystinosis patients nor healthy control had Chiari I malformation. We did not observe any sign of active intracranial hypertension in cystinosis patient. A pineal gland cyst was observed in 4 cystinosis patients and in one healthy control. A vertebral malformation was observed in one cystinosis patient (C1-C2 malformation). We also noted one mild hippocampus atrophy in one cystinosis patient. A significant group effect was found on Evans’ index. More precisely, cystinosis patients had a higher Evans’ index compared to age- and sex-matched healthy controls. Cystinosis patients were significantly more atrophic than age- and sex-matched healthy controls on frontal, parietal, temporal, occipital, corpus callosum and cerebellum. All patients had some degree of parietal atrophy (Fig. 3a), and more than two third of the patients had corpus callosum (Fig. 3b) and cerebellar atrophy (Fig. 3c). Cystinosis patients had significantly more FLAIR hypersignals than age- and sex-matched healthy controls on parietal, occipital, and brain stem/cerebellum (Figs. 3d-h). All patients but one had occipital and medulla oblongata FLAIR hypersignals. Moreover, 8/16 (50%) patients had meningeal FLAIR hypersignals.

**Table 1:** Brain morphometric profile in cystinosis and age- and sex-matched healthy controls

Brain structure	Cystinosis patients (*n* = 16)	Age and sex-matched healthy controls (*n* = 16)	Group comparison
Evans’ Index			*p* < 0.0001
Atrophy			
Frontal	7/16	0/16	*p* < 0.01
Parietal	16/16	1/16	*p* < 0.0001
Temporal	6/16	0/16	*p* < 0.05
Occipital	5/16	0/16	*p* < 0.05
Corpus callosum	11/16	0/16	*p* < 0.0001
Cerebellum	10/16	1/16	*p* < 0.001
FLAIR hypersignals			
Frontal	5/16	1/16	NS
Parietal	5/16	0/16	*p* < 0.05
Temporal	2/16	0/16	NS
Occipital	15/16	3/16	*p* < 0.0001
Brain stem			
*Mesencephalon*	1/16	0/16	NS
*Pons*	9/16	1/16	*p* < 0.005
*Medulla oblongata*	15/16	0/16	*p* < 0.0001
*Around the 4th ventricule including cerebellar pedoncles*	12/16	2/16	*p* < 0.001

**Fig. 4:**
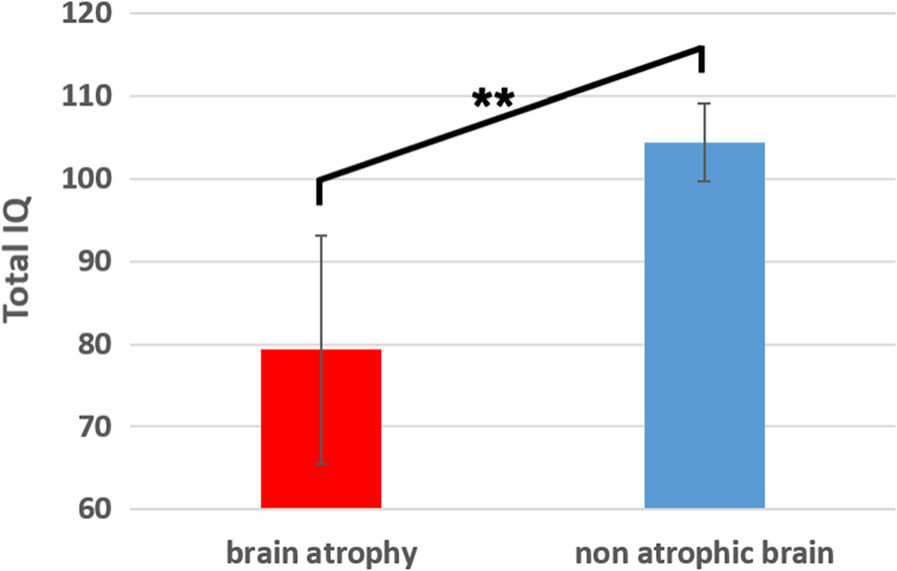
Comparison between atrophic and non-atrophic brain cystinosis patients on the TIQ score

Comparison between atrophic and non-atrophic brain cystinosis patients on the TIQ score showed a significant effect (Fig. 4). More precisely non-atrophic patients had a significantly higher TIQ score (*p* < 0.01). It is interesting to note that the atrophic group mean age was 21.8 years, whereas it was 15 years in the non-atrophic group. Age at cysteamine start was 3.9 years and 2.1 years in the atrophic and non-atrophic group, respectively.

## Discussion

We report here one of the rare series on cystinosis patients including both high resolution neuroimaging and neuropsychological data, as well as their renal status and developmental trajectories. We also perform for the first time a detailed review of literature for both neuroimaging and neuropsychological data in cystinosis patients including all studies published before November 2016 (Table 2–4). Out of the 26 studies we included in the analysis, six (23%) were case-reports and eight series (31%) reported less than 15 cystinosis patients. Among the largest series, it was not specified if cystinosis patients from one study participated also in another one. Fifteen of the studies (58%) did not report the renal condition of the cystinosis patients who were included. Furthermore, 77% did not mention the age of cystinosis patients at cysteamine initiation. Only six studies reported both neuroimaging and neuropsychological data [9, 17, 21, 31–33]. None of them described the age at cysteamine initiation (Table 2). Only three of them had a control group.

**Table 2:** Study characteristics. Descriptive features of the studies included in the review of literature (NA: Non Available). The total number of studies that met the inclusion criteria and were included in the analysis were *k* = 26, comprising 478 patients, with a mean age of 16.1 years, ranging from [1.5 to 47] years. Eighteen studies described neuroimaging data (Table 3) and fifteen studies described neuropsychological data in NC patients (Table 4).

Study name	Number of patients	Mean Age [age range]	Sexe ratio (M/F)	Neurologic disorders associated with NC	Brain MRI	Neuropsycho logical assessment	Age at ESRD (years)	Renal transplants	Age at cysteamine initiation
Cochat, 1985	10	14.2	NA	repeated seizures, tremor, mental retardation, pseudobulbar or pyramidal syndrome	- (TDM)	-	NA	NA	-
		SD 4							
Jonas, 1987	1	25	0	impairment in visual perception, moderate bilateral sensorineural hearing loss, mild peripheral neuropathy,	- (TDM)	+	7	1	NA
		[NA]							
Trauner, 1988	22	11.7	1	16/20 impaired gross and fine motor skills; 11/20 generalized hypotonia; 5/20 intention tremor; 3/20 speech delay; 2/20 microcephaly; 1/20 progressive encephalopathy	-	+	NA	7	10 had not received cysteamine
		[2,9-28,5]							
Nichols, 1990	11	9.8	2.7	NA	+	+	NA	3	NA
		[5,3-19,3]							
Broyer, 1996	7	23	6	neurological complications in 7/26 patients older than 19 years old with 2 forms: - encephalopathie (cerebellar and pyramidal signs, mental deterioration and pseudo-bulbar palsy): 4/7 - stroke-like episode with coma and hemiplegia or milder symptoms	+	-	10.4	7	23,7^b^
		[19-26]							
Ballantyne, 1997	19	8.82	0.9	NA	-	+	NA	NA	NA
		[5,08-25,33]							
Ballantyne, 2000	33	NA	1	NA	-	+	NA	NA	NA
		[5-14]							
Dogulu, 2004	8	22.25	0.6	Documented IntraCranial HyperTension in all patients	+	-	13.2	5	1.9
		[5-47]							
Delgado, 2005	64	8.67	0.94	NA	-	+	-	NA	NA
		[4-16]							
Trauner, 2007	25	NA	1.08	NA	-	+	-	0	20,7 months
		[3,08-8]							
Spilkin, 2007	20	NA		NA	-	+	NA	0	NA
		[4-7]							
Muller, 2008	1	38	0	progressive distal myopathy; cerebellar syndrome regressive under cysteamin	+	-	13	1	29 years
		NA							
Ulmer, 2009	9	9.7	1.25	None	+	+	10.6	4	NA
		[5.3-19.9]							
Berger, 2009	1	29	1	cervical myelopathy and focal seizures	+	-	8	1	20 years
		NA							
Trauner, 2010	52	NA	1.5	NA	+	+	NA	NA	NA
		[2-17] 2 groups (n=26): preacademic [2-5]; school-age [6-17]							
Bava, 2010	24	5.5		none	+	+	NA	NA	NA
		[3-7]							
Besouw, 2010	14	10.5	1.33	NA	-	+	NA	4	1.8
		[6-17]							
Rogers, 2010	6	NA	1	3/6 had intracranial hypertension (age range [19-22])	+	-	NA	3	NA
		[7-22]							
Marquardt, 2013	1	21	0	Posterior Reversible Encephalopathy Syndrome (PRES): generalized seizures, headache, hypertension, vigilance deterioration	+	-	peritoneal dialysis	NA	NA high doses (3g/day)
		NA							
Cazals, 2013	1	24	1	encephalopathy: stroke, then gradually walkind difficulties, cerebellar and frontal pyramidal syndrome	+	-	8	1	NA poor adherence to treatment
		NA							
Neutel, 2013	1	32	0	recurrent ischemic stroke	+	-	12	1	NA
		NA							
Ballantyne, 2013	28	12.16		NA	+	+	NA	8	NA
		[8-17,5]			( n=16)^a^				
Viltz, 2013	46	7.3		NA	-	+	NA	NA	"early / late" treatment
		[3-18]							
Aly, 2014	13	5.9	1.2	NA	+	+	NA (3 were ESRD)	0	NA
		[1,5-12]							
Rao, 2015	53	NA		10/53 had Chiari I malformation, 2 of the 10 were symptomatic	+	-	NA	NA	NA
		[3-18]							
Martin-Begué, 2016	8	9.6	1.67	Intracranial Hypertension in 4/8 (at the age of 6-10 years); 1/4 symptomatic (had a Arnold-Chiari anomaly and enlarged ventricules); 2/4 VPD	+	-	NA	2	NA
		[5-14]							

^a^only in a sub-group (n=16), data from a previous study

^b^cysteamine seemed to be effective on patients with encephalopathy (2/4: complete disappearance, 1/4 partially improved)

In our series, cystinosis patients have a specific neuroanatomical profile, which might help to explain their cognitive profile. Their neuropsychological profile associates visuo-spatial, sensori-motor, and executive functions (including attention) impairment. This is in accordance with previous neuropsychological studies (Table 4). Visuo-spatial processing was shown to be impaired in cystinosis patients in several studies [31, 32, 34–36]. Motor processing, especially fine motor coordination skills [21, 33, 35, 36] and executive functions (including attention deficit, speed of processing, simultaneous processing, cognitive flexibility) were found to be impaired in cystinosis patients [17, 18, 37].

In our series, the neuroanatomical dysmorphic pattern shows cortical and sub-cortical atrophy (especially in parieto-occipital cortex) and FLAIR hypersignals, and is consistent with the neuropsychological difficulties of cystinosis patients (especially parietal atrophy and visuo-spatial impairment) [38]. Moreover, it is unlikely that the brain atrophy could be related to corticosteroids treatment as only a minority of patients received such treatment when the MRI was performed and as it has been shown to affect more specifically the volume of hippocampus [39].

Regarding the neuroimaging studies from the literature, only four studies (22%) included a healthy control group (Table 3). An additional study included a control patient group with another primary renal disease. All MRI studies used a 1.5 T scanner. Some degree of brain atrophy was reported in 72% of the studies [7–10, 12, 13, 17, 21, 22, 31–33, 40]. Two studies tried to score the degree of atrophy [9, 21]. No study reported the atrophy localization in term of brain area. Only one study reported two MRI in the same patient [40]. It is interesting to note that the second one performed eleven years later did not show any progression of the atrophy. White matter anomalies were reported in five studies [7, 25, 31, 32, 41]. Ischemic strokes [7, 10, 11], Chiari malformations [13, 15, 17, 21] and intracranial hypertension [13, 14] were reported in three, four and two studies, respectively.

**Table 3:** Review of the literature on neuroimaging data in cystinosis patients.

Study name	Neurological symptoms	Psychiatric symptoms	Neuroimaging data				Control groups
			number	type	sequence type	abnormalities	
Cochat, 1985	repeated seizures, tremor, mental retardation, pseudobulbar or pyramidal syndrome		10	TDM	-	brain atrophy	10 control patients with another primary renal disease (mean age=11,8 years, SD=3,7)
Jonas, 1987	impairment in visual perception, moderate bilateral sensorineural hearing loss, mild peripheral neuropathy,	depression, lack of motivation, inability to function independently. One year later: confusion with impaired short-term memory	1	TDM	-	cerebral atrophy	no
Nichols, 1990	NA		10	1.5T MRI	T1, T2, DP	subjective cerebral atrophy in 10 of the 11 patients (7: moderate to severe, 3: mild)	no
			1	TDM			
Broyer, 1996	neurological complications in 7 patients with 2 forms: - encephalopathie (cerebellar and pyramidal signs, mental deterioration and pseudo-bulbar palsy): 4/7 - stroke-like episode with coma and hemiplegia or milder symptoms		2	1.5T MRI	T2	moderate brain atrophy, spontaneously regressive T2 hyperintensities in the 2 lenticular nuclei and in the caudate nucleus, a right temporo-occipital area of demyelinization	no
			6	TDM	-	brain atrophy (6/6); multiple areas of mineralization (4/6) including basal ganglia and frontal WM; frontotemporal ischemic stroke (1/6)	
Dogulu, 2004	Documented ICHT (papilledema, CSF opening pressure greater than 200mm of H2O)		7	1.5T MRI	NA	mild diffuse atrophy in 1 patient	no
			1	TDM		none	
Muller, 2008	progressive distal myopathy; cerebellar syndrome regressive under cysteamin	recurrent depressive episodes	2	1,5T MRI	NA	cerebral atrophy (the second MRI performed 11 years later did not reveal any progression of the atrophy)	no
			1	FDG PET		none	
Ulmer, 2009	none		9	1.5T MRI	T1, T2, DTI, ^1^H MR spectroscopy	- 4 mildly or moderately enlarged ventricles, - a mesial slerosis of the hippocampus, - a developmental venous anomaly	no
Berger, 2009	increasing neck pain and left hand and finger numbness; impaired coordination in both upper extremities; dysmetria (L>R), spasticity in both legs, strength deficit (4/5), unable to walk without assistance, pyramidal syndrome, diminished position sense in left , fingers and in left lower extremity, diminished vibratory sense in both lower extremities, impaired in pinprick and temperature sensation on the right side, and focal seizures		1	1,5T MRI	T2, FLAIR	T2 and FLAIR hyperintensities extended throught the brainstem into both internal capsules; small subcortical lesions in both parietal lobes	no
			1	Spinal chord MRI		edematous cord along the entire extent of the cervical cord with contrast-enhancing lesions^a^	
Trauner, 2010	NA		46	1,5T MRI	T1	- 25 N, - 11 mild volume loss, - 5 moderate to severe volume loss, - 5 isolated Chiari I malformation; - No difference in motor coordination between different MRI groups (but very small number of patients per group); No age-related MRI findings	49 controls [2-17] years
Bava, 2010	none		21^b^	1,5T MRI	3DT1, DTI 6 directions	8/24 cortical or central atrophy; significantly decreased FA in the following manually defined ROI: bilateral superior parietal lobule and right inferior parietal lobule, but not in temporal ROI, in cystinosis versus controls; in children older than 5: positive relationship between FA in the RIPL and performance on the Beery VMI test (visuospatial performance); positive correlation between age and FA in the RIPL, LIPL and LSPL in cystinosis patients but not in controls	24 TD age-matched controls
Rogers, 2010	3/6 had intracranial hypertension (age range [19-22]), all were post-renal transplant, 1 marked optic nerve atrophy, 1 papilledema, 2 wide optic nerve sheath diameter measured with a B-scan ultrasound; 3/3 optic nerve sheath fenestration, 1/3 VPD		3	1,5T MRI	NA	N	no
Marquardt, 2013	Posterior Reversible Encephalopathy Syndrome (PRES): generalized seizures, headache, hypertension, vigilance deterioration		1	1,5T MRI	FLAIR, ADC, contrast enhanced MR angiogram	parieto-occipital and fronto-parietal mild oedematous swelling	no
Cazals, 2013	encephalopathy: stroke, then gradually walkind difficulties, cerebellar and frontal pyramidal syndrome		1	1,5T (and 3T 3 years later)	Diffusion, FLAIR, T2*	diffuse atrophy, multiple ischemic lesions in the semi-oval centre, T2 hyperintensity in the periventricular areas, a few frontal sub-cortical T2* hypointensity and slight uptake of contrast in the pervascular spaces in the fronto-parietal WM and central grey nuclei	no
				CT angiography		N	
Neutel, 2013	sudden onset of speech and gait disturbance		1	1,5T MRI	DWI, angioMRI	recurrent strokes caused by intracranial arterial stenosis (right internal carotid artery stenosis and right middle cerebral artery stenosis)	no
Ballantyne, 2013	NA		16	1,5T MRI	NA	12: N; 1: Chiari I malformation; 3: moderate cortical volume loss for age	MRI results from an earlier study
Aly, 2014	NA		13	1,5T MRI	NA	7/13 cortical brain atrophy, dysmyelination	13 age- and sex-matched healthy controls
Rao, 2015	10/53 had Chiari I malformation, 2 of the 10 were symptomatic		53	1,5T MRI	3DT1	10/53 (18,9%) Chiari I malformation (>5mm) or tonsillar ectopia (3-5mm), 1 syrinx, 1 syringohydromyelia	120 healthy controls: 2 (1,6%) Chiari I or tonsillar ectopia
Martin-Begué, 2016	Intracranial Hypertension in 4/8 with papilledema and confirmed high CSF pressure (at the age of 6-10 years); 1/4 symptomatic (had a Arnold-Chiari anomaly and enlarged ventricules); 2/4 VPD		4	1,5T MRI	NA	1 Arnold-Chiari and enlarged ventricules; 1 great distension of the perioptic subarachnoid space; 2N	

^a^a spinal cord biopsy was performed: chronic active demyelinating myelitis with lymphocytic vasculitis, atypical astrocytes, and microglial clusters ; CSF: lymphocytic pleocytosis, elevated protein and increased IgG synthesis

^b^2 exclusions for motion artifacts, 1 for susceptibility artifact

In our series, the mean total IQ is within the normal range, as it has also been previously reported [9, 16, 18, 20, 22, 31, 33, 34, 36, 37]. Two of our patients have multiple specific learning disorder syndrome, and one has pre-dementia. In the literature, intelligence assessment was performed using Stanford Binet scale [9, 16, 20, 31, 34] or Wechsler scale [18, 21, 22, 32, 33, 35–37], Table 4. Mean IQ in cystinosis patient groups was reported in 10 out of these 13 studies and was within the normal range in the 10 studies. It is nevertheless interesting to note that the mean IQ distribution was as follows: in six studies it was within the average range (between 90 and 109), in 3 within the low average range (between 80 and 89) and in one it was borderline (between 70 and 79), Table 4. In the latter study, cystinosis patients had also lower socioeconomic status, which might have contributed to this result [31]. Another study included a very interesting control group despite its small size, with unaffected siblings of the cystinosis patients, and did not show any difference on composite IQ (Stanford Binet scale) between the two groups [20]. Several studies reported discrepancies between performance (being lower) and verbal IQ [18, 21, 31, 33, 36, 37]. Further studies are needed to investigate if verbal IQ, despite being within the normal range and higher than performance IQ, is lower than in controls as suggested in some studies [31, 32, 35].

**Table 4:** Review of the literature on neurocognitive data in cystinosis patients. (VMI: Visual Motor Integration). Regarding the neuropsychological studies, 11/15 (73.3%) included a control group.

Study name	Control group	Neuropsychological assessment												
		Stanford Binet			Wechsler scale								Other tests	Results
		Number of patients	Composite IQ		Test name	Number of patients	Total IQ		Verbal IQ		Performance IQ		Test name	
			mean	SD			mean	SD	mean	SD	mean	SD		
Jonas, 1987 [22]					WAIS-III	1	89	NA	90	NA	90	NA		This patient never attended school, but she received home-bound teaching and obtained a high school diploma.
Trauner, 1988 [20]	unaffected sibling control group (*n* = 12); chronic renal failure contrôle group (*n* = 8)	18/22 (selection of 5 subtests)	97.6	9.8										No difference from their siblings on composite IQ; significantly higher than the scores from patients with renal failure; bead memory subtest significantly below the norm in cystinosis patietns (*p* < 0,001): deficit in short term visual memory
Nichols, 1990 [9]	no	8/11 (abbreviated version)	101.4	14.3									Benton Visual Retention test (*n* = 11); Beery test of visual motor integration (*n* = 11)	Children with the greatest degree of atrophy had the poorest performance on all cognitive tests, however only the relationship beween atrophy and short-term memory approached significance (*p* = 0.06); short-term memory was assessed with 2 subtest: bead memory (visual) and memory for sentences (auditory): poorer performance on visual memory
Ballantyne, 1997 [16]	age-, sex- and IQ matched healthy control group (*n* = 19)	19	108	10									Wide Range Achievement Test-Revised (*n* = 19)	All patients had IQ within the normal range; performed significantly more poorly on arithmetic sub-score compared to controls (*p* = 0,001), trends on spelling (*p* = 0,085); no deterioration of function with age; all but 2 were grade-appropriate for their age (one was a year behind, the other one year advanced)
Ballantyne, 2000 [34]	healthy control group (*n* = 108)	33	102.65	14.2									Visuospatial test^a^; visuoperceptual tests^b^; visual scanning tests^c^	Mean IQ within the normal range; All comparisons on the other tests were based on raw scores and revealed: impairment in spatial processing (extrapersonal orientation, mental rotation, short-term memory of spatial location) whereas perceptual processing was largely intact
Delgado, 2005 [42]	healthy control group (*n* = 101) and cystic fibrosis patients (*n* = 21)												Achenbach Child Behavior CheckList (*n* = 64): Total score, Internalizing and Externalizing behavior	The group means for all scales were within the normal range, but rates of clinically significant scores were higher on the total problems summary scale and the internalizing problems summary scale; significant differences compared to controls on: social problems (immature behavior), somatic complaints, attention problems, and aggressive behaviors
Trauner, 2007 [35]	age-matched control group (*n* = 25)				WPPSI-III, WISC-III, WISC-IV	25	NA	NA	93.4	12.73	NA	NA	Gollin Incomplete Figures (*n* = 25), Motor-Free Visual Perception Test (*n* = 25), Beery-Buktenica Developmental Test of VMI 5th ed. (*n* = 25) including visual perception supplemental test; K-ABC spatial memory sub-test (*n* = 25), Spatial Relations Test of the Woodcock-Johnson Psycho-educational battery 3rd ed. (*n* = 25)	TIQ and PIQ were not reported; significant lower VIQ in NC patients compared to controls (although VIQ was within the normal range in NC patients); a significant impairment in visual spatial and visual motor skills was found in young children with NC, whereas no significant difference on visual perceptual tests.
Spilkin, 2007 [18]	20 age-matched typically developped controls				WPPSI-III	20	90.85	10.8	96.25	10.7	89	12.9		Mean TIQ at the low end of the average range, average verbal abilities, low average non-verbal abilities and processing speed, discrepancy between verbal and non-verbal abilities
Ulmer, 2009 [33]	no				WISC-III	7	92	[71-105]	93	[76-118]	90	[68-97]	Zurich Neuromotor assessment (*n* = 8); Child Behavior Checklist (*n* = 8); the TNO-AZL quality of life questionnaire (child- and parent-forms, *n* = 7); K-ABC (*n* = 2, included in the mean of IQ reported using WISC-III)	Total IQ was significantly lower in NC patients compared to controls. Verbal IQ was significantly higher than performance (*p* = 0,03). Motor performance was significantly below the norm. The patients’ QOL was normal for six of seven dimensions, whereas parents’ QOL rated only 3 of seven dimensions as normal. Psychosocial ajustment was signficantly impaired in NC patients.
Trauner, 2010 [21]	49 controls [2-17] years				WPPSI-III or WISC-III or WAIS-III, WISC-IV	52			95.04	+/−12,5	89.27	+/− 14,7	motor coordination supplemental test of Beery test of visual motor integration (*n* = 52)	In the younger group (preacademic): no significant differences between NC patients and controls on VIQ, but significantly lower PIQ; in the older group (school age): both VIQ and PIQ were significantly lower in NC patients; persistant non progressive, fine-motor coordination deficit in NC patients
Bava, 2010 [32]	24 TD age-matched controls				WISC-R or WISC III	24			95	+/−12,2	90	+/−12	Beery test of VMI (*n* = 24); Spatial Relations Test of the Woodcock-Johnson Psycho-educational battery third edition (*n* = 24)	NC patients had lower scores than controls on both visuospatial functionning measures and on both measures of intellectual functioning (PIQ and VIQ)
Besouw, 2010 [37]	no				WISC-III (*n* = 13) and WAIS-III (*n* = 1)	14	87	[60-132]	95	[60-125]	87	[65-130]	Beery test of visual motor integration (*n* = 14); Stroop Color-Word Interference Test (*n* = 11); Bourdon-Vos test (*n* = 13); Rey-Osterrieth complex figure (*n* = 12); computerized drawing task (*n* = 14); Child BehaviorChecklist (*n* = 14)	Median Total IQ was below the average of normal population in NC patients with a discrepancy between verbal and performance IQ; 50% of NC patients scored poorly on visual-motor integration (VMI < 85), 69% had sustained attention impairment, none had a good visual memory score, only one had good planning skills and two (14%) had a good score on processing speed. No significant difference was noted on behavioural and emotional functioning.
Ballantyne, 2013 [17]	24 age-matched healthy controls (mean age:12,1 years, [8,1-17,8]												Stroop Color-Word Interference Test (*n* = 28); Delis-Kaplan executive function system (D-KEFS, *n* = 28); Behavior Rating Inventory of Excecutive Function (BRIEF questionnaire, *n* = 28)	Higher incidence of executive dysfunction in NC patients (including attention, initiation, motor speed, fluency, simultaneous processing, speed of processing, cognitive flexibility, inhibiting prepotent responses, abstract thought)
Viltz, 2013 [36]	age-matched control group (*n* = 85)				WISC-III	46 (with 2 groups: early *n* = 32 / late treatment *n* = 14)	88.5	early ttt: 94(+/−12,5)/late ttt: 83(+/− 15,1)	93.4	early ttt: 98,2(+/−11,1)/late ttt: 88,6(+/−15,2)	87.2	early ttt: 92,7(+/−15,3)/late ttt: 81,6(+/−13,2)	Beery test of VMI (*n* = 41); Spatial Relations Test of the Woodcock-Johnson Psycho-educational battery third edition (*n* = 40)	NC patients scored significantly lower on visuomotor skills compared with controls; NC patients scored lower on IQ measures and visuo-spatial skills when treated at or after age 2 years compared with NC patients treated before the age of 2 years and controls.
Aly, 2014 [31]	13 age- and sex-matched healthy controls	13 (arabic version, second edition)	78.9										Child Behavior Checklist (*n* = 13); Porteus Maze Test (Visuospatial ability and visuomotor coordination, *n* = 13)	Total IQ was significantly lower in NC patients compared to controls; both verbal and non verbal and non verbal abilities being impaired; the non verbal abilities were lower (but did not reach statistical significance); visuospatial abilities were significantly lower compared to visual perception; NC patients had more behavioral problems compared to healthy controls; NC patients had lower socioeconomic status in this study.

^a^locomotor maze (*n* = 19), Luria-Nebraska visuospatial test (*n* = 25), K-ABC spatial memory (*n* = 30)

^b^Gollin incomplete figures (*n* = 33), Children’s embedded figures test (*n* = 25)

^c^cancellation (cross out target forms, *n* = 24)

Interestingly, in our series, we found a significant correlation between the degree of brain atrophy and the Total IQ score. Non-atrophic cystinosis patients had significantly higher IQ compared to atrophic cystinosis patients. This finding is in accordance with other studies which found poorest performance in cystinosis patients with the greatest degree of atrophy but did not reach significance, likely due to the small number of patients included [9]. Furthermore, in our series, the memory assessment reveals impaired working memory, but does not show any dissociation between visual and verbal memory. Two other studies suggested that visual memory was more impaired than auditory memory in cystinosis patients [9, 20]. But the tests we used were different (they used two sub-tests from the Standford-Binet test (bead memory and memory for sentences subtests), whereas we chose to use a battery focused on assessing memory skills). One study suggested that cystinosis patients performed significantly more poorly on arithmetic sub-score compared to controls [16]. Some studies reported more psychosocial adjustment issues in cystinosis patients [31, 33, 42], whereas another did not find any emotional nor behavioural dysfunction [37].

The developmental trajectories of cystinosis patients reveal that they may present with motor delay (walk after 18 months), and/or first words utterance delay. Their renal pathology (proximal tubulopathy) might also interfere with the milestones of psychomotor development in cystinosis patients. Almost one third of the cystinosis patients had to repeat a grade, but only one patient went to specialized education.

We found a significant correlation between the age at start of cysteamine and Total IQ: the sooner cysteamine was started, the higher the IQ was. This underscores the need of early diagnosis and appropriate treatment. Given the limited number of children older than 4 years, this result will need to be confirmed on a larger cohort. However, this is in accordance with another study showing that cystinosis patients treated before the age of 2 years had better outcome compared to cystinosis patients treated after the age of 2 [36]. Furthermore, in our study cysteamine treatment was started significantly later in the atrophic group compared to the non-atrophic cystinosis patient group.

3DT1 and FLAIR sequences seem to be sensitive to detect brain anomalies in cystinosis patients. It is interesting to note that all cystinosis patients had some degree of cerebral atrophy in parietal region. This is concordance with a study showing DTI abnormalities in cystinosis patients with bilateral decrease in FA in the inferior and superior parietal lobules [32]. Based on neuropathological studies, we might assume that the brain anomalies we detected in cystinosis patients are likely related to both cystine accumulation, with cystine crystals occurring in oligodendrocytes and leading to inflammation, and vasculopathy affecting small and medium sized blood vessels [11, 22, 24, 25]. We also report in this study for the first time FLAIR hypersignals located on medulla oblongata and around the 4th ventricule including cerebellar pedoncles. The longitudinal follow-up of these patients will help to understand the significance of these images. Moreover, we observed FLAIR meningeal hypersignal in 50% of the patients. This is interesting as Jonas et al. reported thickened dura and leptomeninges on autopsy examination [22].

Our study has some limitations. We did not include another control group, such as chronic kidney disease (CKD), to be sure that the differences we observed were not related to renal disease. In the literature, earlier studies in CKD young children reported up to 65% of developmental delay and 49% of “encephalopathy” in young children with CKD [43, 44]. However, advances such as avoidance of aluminium (which led to aluminium-induced neurotoxicity secondary to CKD treatment), improved nutrition, improved anemia control (with erythropoietin), have significantly decrease the prevalence of developmental delay in CKD. Articles that are more recent showed that children with CKD had median scores for almost all cognitive measures within the normal range [45–48]. More precisely, a mean difference of − 10.5 was noticed between CKD children and the general population [45, 47, 49, 50]. A significant negative correlation was found between CKD stages and Wechsler IQ test [51]. Longer CKD duration has been associated with poorer performance on attention regulation and inhibitory control [52]. However, no effect of eGFR was observed on attention test performance [52] (Mendley et al., 2014). Executive functions seemed altered in CKD patients, especially initiation and sustaining executive function domains [47, 49]. 21 to 41% of CKD patients scored at least one SD below the mean on measures of academic achievement [47], with the greatest deficit in mathematics [45, 53] and were at higher risk for grade retention and absenteeism [54]. However, no major neurocognitive deficits was observed in mild to moderate CKD patients, with eGFR of 30 to 90 ml/mn/1.73 m^2^ [47, 48]. Predialysis patients and dialysis patients are likely to exhibit cognitive impairment [55, 56]. Verbal, Performance and Full Score IQs of patients with ESRD (eGFR< 18 ml/mn/1.73m^2^) were significantly lower than the IQs of sibling controls [57].

Interestingly, as 76% of our cohort had eGFR above 30 ml/mn/1.73m^2^, we could expect mild cognitive impairment, consistent with the IQ scores we observed, and executive dysfunction (including attention) to be related to CKD. The visuo-spatial impairment we found seem to be more likely specific to cystinosis patients. No such dissociation between Verbal and Performance IQ was observed in other large (*n* = 368 children) CKD series [47], with the exception of small series including cystinosis patients [46].

Brain atrophy has been reported in 12 to 23% of children with ESDR [58]. It is interesting to note that research focused on diseases at greater risk for cerebral dysfunction, including cystinosis, Lowe syndrome and congenital nephrotic syndrome [43, 58]. More white matter disease (using diffusion tensor imaging) has been described in the anterior than posterior parts of the brain in adult hemodialysis patients compared to controls, leading to suggest that CKD may result in a phenotype consistent with accelerated aging [59, 60]. Kidney function biomarkers (eGFR and urine albumin to creatinine ratio) were associated with MRI brain changes, even after accounting for vascular risk factor, in adults (> 45 years) with oversampling of moderate to severe CKD [61]. Lower eGFR was associated with greater white matter hyperintensities burden increased odds of cortical infarction, and worsening diffusion changes throughout the brain [61]. The impact on grey matter is minimal in mild to moderate stages of CKD, and becomes significant in ESRD [61–63].

If CKD might have contributed to cerebral atrophy we observed in our cohort, it is more likely that cystinosis has a direct impact on the brain atrophy. Furthermore, in cystinosis patients, the parieto-occipital atrophy was consistent with the visuo-spatial specific impairment.

## Conclusion

Long-lasting longitudinal studies with repeated brain MRI, repeated neuropsychological testing, and objective hand grip strength assessment (JAMAR) in cystinosis patients are necessary to further understand how long-term neurological complication may occur. It would be very interesting to compare cystinosis patients and CKD patients in such longitudinal studies. Given that cystinosis patients are at risk of developing stroke, primary prevention of stroke could also been discussed in these patients. We suggest that a systematic neuropsychological assessment might be helpful in cystinosis children, in order to help them with appropriate academic accommodation and rehabilitation as soon as possible.

## Supplementary information

**Additional file 1: Figure S1.** Dissociation between General Memory and Working memory skills in cystinosis patients.

## Data Availability

All data analysed during this study are included in this published article and its supplementary information files.

## Abbreviations

3 T: 3 Tesla
ACD: Acid Citrate Dextrose
BMI: Body Mass Index
CKD: Chronic Kidney Diease
ESRD: End-Stage Renal Disease
FOV: Field Of View
GE: General Electrics
HC: Head Circumference
IQ: Intelligence Quotient
MRI: Magnetic Resonance Imaging
PRI: Perceptual Reasoning Index
PSI: Processing Speed Index
SD: Standard Deviation
TE: Time of Echo
TFE: Turbo Field Echo
TI: Time of Inversion
TR: Time of Repetition
VCI: Verbal Comprehension Index
WBC: White Blood Cell
WMI: Working Memory Index

## References

[1] Gahl WA, Thoene JG, Schneider JA (2002). Cystinosis. N Engl J Med.

[2] Emma F, Nesterova G, Langman C, Labbe A, Cherqui S, Goodyer P, Janssen MC, Greco M, Topaloglu R, Elenberg E, Dohil R, Trauner D, Antignac C, Cochat P, Kaskel F, Servais A, Wuhl E, Niaudet P, Van't Hoff W, Gahl W, Levtchenko E (2014). Nephropathic cystinosis: an international consensus document. Nephrol Dial Transplant.

[3] Broyer M, Guillot M, Gubler MC, Habib R (1981). Infantile cystinosis: a reappraisal of early and late symptoms. Adv Nephrol Necker Hosp.

[4] Brodin-Sartorius A, Tete MJ, Niaudet P, Antignac C, Guest G, Ottolenghi C, Charbit M, Moyse D, Legendre C, Lesavre P, Cochat P, Servais A (2012). Cysteamine therapy delays the progression of nephropathic cystinosis in late adolescents and adults. Kidney Int.

[5] Gahl WA, Balog JZ, Kleta R (2007). Nephropathic cystinosis in adults: natural history and effects of oral cysteamine therapy. Ann Intern Med.

[6] Sonies BC, Almajid P, Kleta R, Bernardini I, Gahl WA (2005). Swallowing dysfunction in 101 patients with nephropathic cystinosis: benefit of long-term cysteamine therapy. Medicine (Baltimore).

[7] Broyer M, Tete MJ, Guest G, Bertheleme JP, Labrousse F, Poisson M (1996). Clinical polymorphism of cystinosis encephalopathy. Results of treatment with cysteamine. J Inherit Metab Dis.

[8] Cochat P, Drachman R, Gagnadoux MF, Pariente D, Broyer M (1986). Cerebral atrophy and nephropathic cystinosis. Arch Dis Child.

[9] Nichols SL, Press GA, Schneider JA, Trauner DA (1990). Cortical atrophy and cognitive performance in infantile nephropathic cystinosis. Pediatr Neurol.

[10] Cazals X, Lauvin MA, Favelle O, Domengie F, Nivet H, Cottier JP (2013). Cystinosis encephalopathy: MRI perivascular enhancement with micronodular T2* hypointensity. Diagn Interv Imaging.

[11] Neutel D, Geraldes R, Pereira P, Gomes da Costa A, Pimentel J, Melo TP e (2013). Recurrent ischemic stroke in an adult with cystinosis: a clinical-pathological case. J Stroke Cerebrovasc Dis.

[12] Dogulu CF, Tsilou E, Rubin B, Fitzgibbon EJ, Kaiser-Kupper MI, Rennert OM, Gahl WA (2004). Idiopathic intracranial hypertension in cystinosis. J Pediatr.

[13] Martín-Begué N, Alarcón S, Wolley-Dod C, Lara LE, Madrid Á, Cano P, Del Toro M, Ariceta G. Intracranial Hypertension in Cystinosis Is a Challenge: Experience in a Children's Hospital. JIMD Rep. 2017;35:17–22. 10.1007/8904_2016_18 PMC5585107 27858370

[14] Rogers DL, McGregor ML (2010). Increased intracranial pressure in patients with cystinosis. J Pediatr Ophthalmol Strabismus.

[15] Rao KI, Hesselink J, Trauner DA (2015). Chiari I malformation in Nephropathic Cystinosis. J Pediatr.

[16] Ballantyne AO, Scarvie KM, Trauner DA (1997). Academic achievement in individuals with infantile nephropathic cystinosis. Am J Med Genet.

[17] Ballantyne AO, Spilkin AM, Trauner DA (2013). Executive function in nephropathic cystinosis. Cogn Behav Neurol.

[18] Spilkin AM, Ballantyne AO, Babchuck LR, Trauner DA (2007). Non-verbal deficits in young children with a genetic metabolic disorder: WPPSI-III performance in cystinosis. Am J Med Genet B Neuropsychiatr Genet.

[19] Trauner D (2017). Neurocognitive complications of Cystinosis. J Pediatr.

[20] Trauner DA, Chase C, Scheller J, Katz B, Schneider JA (1988). Neurologic and cognitive deficits in children with cystinosis. J Pediatr.

[21] Trauner DA, Williams J, Ballantyne AO, Spilkin AM, Crowhurst J, Hesselink J (2010). Neurological impairment in nephropathic cystinosis: motor coordination deficits. Pediatr Nephrol.

[22] Jonas AJ, Conley SB, Marshall R, Johnson RA, Marks M, Rosenberg H (1987). Nephropathic cystinosis with central nervous system involvement. Am J Med.

[23] Levine S, Paparo G (1982). Brain lesions in a case of cystinosis. Acta Neuropathol.

[24] Vogel DG, Malekzadeh MH, Cornford ME, Schneider JA, Shields WD, Vinters HV (1990). Central nervous system involvement in nephropathic cystinosis. J Neuropathol Exp Neurol.

[25] Berger JR, Dillon DA, Young BA, Goldstein SJ, Nelson P (2009). Cystinosis of the brain and spinal cord with associated vasculopathy. J Neurol Sci.

[26] Maurice T, Hippert C, Serratrice N, Dubois G, Jacquet C, Antignac C, Kremer EJ, Kalatzis V (2009). Cystine accumulation in the CNS results in severe age-related memory deficits. Neurobiol Aging.

[27] Kamoun, P., C. Vianey-Saban, J. Aupetit, S. Boyer and B. Chadefaux-Vekemans (1999). "Measurement of cystine in granulocytes and leucocytes: methodological aspects. ." Cystinosis. Broyer M, editor. Amsterdam: Elsevier: 86–92.

[28] Piraud M, Vianey-Saban C, Bourdin C, Acquaviva-Bourdain C, Boyer S, Elfakir C, Bouchu D (2005). A new reversed-phase liquid chromatographic/tandem mass spectrometric method for analysis of underivatised amino acids: evaluation for the diagnosis and the management of inherited disorders of amino acid metabolism. Rapid Commun Mass Spectrom.

[29] Schwartz GJ, Munoz A, Schneider MF, Mak RH, Kaskel F, Warady BA, Furth SL (2009). New equations to estimate GFR in children with CKD. J Am Soc Nephrol.

[30] Selistre L, Rabilloud M, Cochat P, de Souza V, Iwaz J, Lemoine S, Beyerle F, Poli-de-Figueiredo CE, Dubourg L (2016). Comparison of the Schwartz and CKD-EPI equations for estimating glomerular filtration rate in children, adolescents, and adults: a retrospective cross-sectional study. PLoS Med.

[31] Aly R, Makar S, El Bakri A, Soliman NA (2014). Neurocognitive functions and behavioral profiles in children with nephropathic cystinosis. Saudi J Kidney Dis Transpl.

[32] Bava S, Theilmann RJ, Sach M, May SJ, Frank LR, Hesselink JR, Vu D, Trauner DA (2010). Developmental changes in cerebral white matter microstructure in a disorder of lysosomal storage. Cortex.

[33] Ulmer FF, Landolt MA, Vinh RH, Huisman TA, Neuhaus TJ, Latal B, Laube GF (2009). Intellectual and motor performance, quality of life and psychosocial adjustment in children with cystinosis. Pediatr Nephrol.

[34] Ballantyne AO, Trauner DA (2000). Neurobehavioral consequences of a genetic metabolic disorder: visual processing deficits in infantile nephropathic cystinosis. Neuropsychiatry Neuropsychol Behav Neurol.

[35] Trauner DA, Spilkin AM, Williams J, Babchuck L (2007). Specific cognitive deficits in young children with cystinosis: evidence for an early effect of the cystinosin gene on neural function. J Pediatr.

[36] Viltz L, Trauner DA (2013). Effect of age at treatment on cognitive performance in patients with cystinosis. J Pediatr.

[37] Besouw MT, Hulstijn-Dirkmaat GM, van der Rijken RE, Cornelissen EA, van Dael CM, Vande Walle J, Lilien MR, Levtchenko EN (2010). Neurocognitive functioning in school-aged cystinosis patients. J Inherit Metab Dis.

[38] Marshall JC, Fink GR (2001). Spatial cognition: where we were and where we are. Neuroimage.

[39] Uno H, Eisele S, Sakai A, Shelton S, Baker E, DeJesus O, Holden J (1994). Neurotoxicity of glucocorticoids in the primate brain. Horm Behav.

[40] Muller M, Baumeier A, Ringelstein EB, Husstedt IW (2008). Long-term tracking of neurological complications of encephalopathy and myopathy in a patient with nephropathic cystinosis: a case report and review of the literature. J Med Case Rep.

[41] Marquardt L, Kuramatsu JB, Roesch J, Engelhorn T, Huttner HB (2013). Posterior reversible encephalopathy syndrome in cystinosis. Clin Neurol Neurosurg.

[42] Delgado G, Schatz A, Nichols S, Appelbaum M, Trauner D (2005). Behavioral profiles of children with infantile nephropathic cystinosis. Dev Med Child Neurol.

[43] Gipson DS, Wetherington CE, Duquette PJ, Hooper SR (2004). The nervous system and chronic kidney disease in children. Pediatr Nephrol.

[44] Polinsky MS, Kaiser BA, Stover JB, Frankenfield M, Baluarte HJ (1987). Neurologic development of children with severe chronic renal failure from infancy. Pediatr Nephrol.

[45] Chen K, Didsbury M, van Zwieten A, Howell M, Kim S, Tong A, Howard K, Nassar N, Barton B, Lah S, Lorenzo J, Strippoli G, Palmer S, Teixeira-Pinto A, Mackie F, McTaggart S, Walker A, Kara T, Craig JC, Wong G (2018). Neurocognitive and educational outcomes in children and adolescents with CKD: a systematic review and meta-analysis. Clin J Am Soc Nephrol.

[46] Falger J, Latal B, Landolt MA, Lehmann P, Neuhaus TJ, Laube GF (2008). Outcome after renal transplantation. Part I: intellectual and motor performance. Pediatr Nephrol.

[47] Hooper SR, Gerson AC, Butler RW, Gipson DS, Mendley SR, Lande MB, Shinnar S, Wentz A, Matheson M, Cox C, Furth SL, Warady BA (2011). Neurocognitive functioning of children and adolescents with mild-to-moderate chronic kidney disease. Clin J Am Soc Nephrol.

[48] Hooper SR, Gerson AC, Johnson RJ, Mendley SR, Shinnar S, Lande MB, Matheson MB, Gipson DS, Morgenstern B, Warady BA, Furth SL (2016). Neurocognitive, social-behavioral, and adaptive functioning in preschool children with mild to moderate kidney disease. J Dev Behav Pediatr.

[49] Gipson DS, Hooper SR, Duquette PJ, Wetherington CE, Stellwagen KK, Jenkins TL, Ferris ME (2006). Memory and executive functions in pediatric chronic kidney disease. Child Neuropsychol.

[50] Moser JJ, Veale PM, McAllister DL, Archer DP (2013). A systematic review and quantitative analysis of neurocognitive outcomes in children with four chronic illnesses. Paediatr Anaesth.

[51] Youssef DM, Mohamed AH, Attia WMK, Mohammad FF, El Fatah NRA, El-Shal AS (2018). Cerebral metabolic alterations and cognitive dysfunction in children with chronic kidney disease using magnetic resonance spectroscopy and Wechsler intelligence scale. Nephrology (Carlton).

[52] Mendley SR, Matheson MB, Shinnar S, Lande MB, Gerson AC, Butler RW, Warady BA, Furth SL, Hooper SR (2015). Duration of chronic kidney disease reduces attention and executive function in pediatric patients. Kidney Int.

[53] Abdel-Kader K, Dew MA, Bhatnagar M, Argyropoulos C, Karpov I, Switzer G, Unruh ML (2010). Numeracy skills in CKD: correlates and outcomes. Clin J Am Soc Nephrol.

[54] Duquette PJ, Hooper SR, Wetherington CE, Icard PF, Gipson DS (2007). Brief report: intellectual and academic functioning in pediatric chronic kidney disease. J Pediatr Psychol.

[55] Pi HC, Xu YF, Xu R, Yang ZK, Qu Z, Chen YQ, Liu GL, Dong J (2016). Cognitive impairment and structural neuroimaging abnormalities among patients with chronic kidney disease. Kidney Blood Press Res.

[56] Vanderlinden JA, Ross-White A, Holden R, Shamseddin MK, Day A, Boyd JG (2019). Quantifying cognitive dysfunction across the spectrum of end-stage kidney disease: a systematic review and meta-analysis. Nephrology (Carlton).

[57] Bawden HN, Acott P, Carter J, Lirenman D, MacDonald GW, McAllister M, McDonnell MC, Shea S, Crocker J (2004). Neuropsychological functioning in end-stage renal disease. Arch Dis Child.

[58] Gipson DS, Duquette PJ, Icard PF, Hooper SR (2007). The central nervous system in childhood chronic kidney disease. Pediatr Nephrol.

[59] Chiu YL, Tsai HH, Lai YJ, Tseng HY, Wu YW, Peng YS, Chiu CM, Chuang YF (2019). Cognitive impairment in patients with end-stage renal disease: accelerated brain aging?. J Formos Med Assoc.

[60] Drew DA, Koo BB, Bhadelia R, Weiner DE, Duncan S, la Garza MM, Gupta A, Tighiouart H, Scott T, Sarnak MJ (2017). White matter damage in maintenance hemodialysis patients: a diffusion tensor imaging study. BMC Nephrol.

[61] Vemuri P, Knopman DS, Jack CR, Lundt ES, Weigand SD, Zuk SM, Thostenson KB, Reid RI, Kantarci K, Slinin Y, Lakshminarayan K, Davey CS, Murray A (2017). Association of Kidney Function Biomarkers with brain MRI findings: the BRINK study. J Alzheimers Dis.

[62] Murea M, Hsu FC, Cox AJ, Hugenschmidt CE, Xu J, Adams JN, Raffield LM, Whitlow CT, Maldjian JA, Bowden DW, Freedman BI (2015). Structural and functional assessment of the brain in European Americans with mild-to-moderate kidney disease: diabetes heart study-MIND. Nephrol Dial Transplant.

[63] Qiu Y, Lv X, Su H, Jiang G, Li C, Tian J (2014). Structural and functional brain alterations in end stage renal disease patients on routine hemodialysis: a voxel-based morphometry and resting state functional connectivity study. PLoS One.

