# Supplementary material for: Neuropsychological and neuroanatomical phenotype in 17 patients with cystinosis
Source: Orphanet J Rare Dis. 2020 Feb 26;15:59. doi: 10.1186/s13023-019-1271-6 (PMC7045592; doi:10.1186/s13023-019-1271-6)

Supplementary Figure 1 : Dissociation between General Memory and Working memory skills in cystinosis patients.

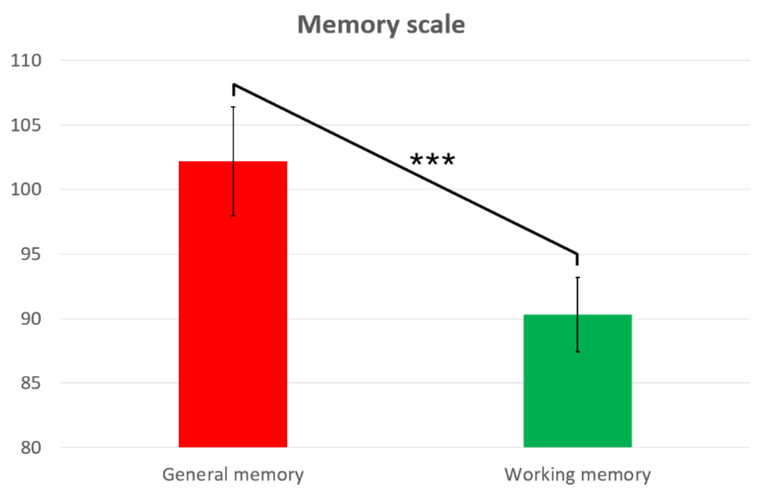

Supplement: Supplementary file 1 — Additional file 1: Figure S1. Dissociation between General Memory and Working memory skills in cystinosis patients. [file 13023_2019_1271_MOESM1_ESM.pdf]
